# Spino-Olivary Projections in the Rat are Natomically Separate From Postsynaptic Dorsal Column Projections

**DOI:** 10.1002/cne.23527

**Published:** 2013-12-20

**Authors:** Charlotte R Flavell, Nadia L Cerminara, Richard Apps, Bridget M Lumb

**Affiliations:** 1 School of Physiology and Pharmacology, University of BristolBristol, UK

**Keywords:** cerebellum, inferior olive, gracile, tracer

## Abstract

The gracile nucleus (GN) and lateral part of rostral dorsal accessory olive (rDAO) are important relays for indirect, postsynaptic dorsal column, and direct ascending pathways, respectively, that terminate as climbing fibers in the “hindlimb-receiving” parts of the C1 and C3 zones in the cerebellar cortex. While the spinal cells of origin of that project to GN and rDAO are from largely separate territories in the spinal cord, previous studies have indicated that there could be an area of overlap between these two populations in the medial dorsal horn. Given the access of these two ascending tracts to sensory (thalamic) versus sensorimotor (precerebellar) pathways, the present study therefore addresses the important question of whether or not individual neurons have the potential to contribute axons to both ascending pathways. A double-fluorescent tracer strategy was used in rats (red Retrobeads and Fluoro-Ruby or green Retrobeads and Fluoro-Emerald) to map the spatial distribution of cells of origin of the two projections in the lumbar spinal cord. The two pathways were found to receive input from almost entirely separate territories within the lumbar cord (levels L3–L5). GN predominantly receives input from lamina IV, while rDAO receives its input from three cell populations: medial laminae V–VI, lateral lamina V, and medial laminae VII–VIII. Cells that had axons that branched to supply both GN and rDAO represented only about 1% of either single-labeled cell population. Overall, the findings therefore suggest functional independence of the two ascending pathways. J. Comp. Neurol. 522:2179–2190, 2014. © 2013 Wiley Periodicals, Inc.

To make its vital contribution to movement control, the cerebellum is dependent on sensory signals from skin, joints, and muscles that are conveyed by a complex array of ascending pathways. A major component of these paths includes a precerebellar synaptic relay within the inferior olive. The latter is the sole source of climbing fiber afferents that terminate in longitudinally organized zones within the contralateral cerebellar cortex (for review, see, e.g., Voogd and Bigaré, 1980; Apps and Hawkes, 2009). The importance of the olivo-cerebellar projection is emphasized by the fact that partial or complete olivary ablation leads to motor deficits that resemble those that follow cerebellectomy (e.g., Murphy and O’Leary, 1971; Seoane et al., 2005).

Of the various paths that transmit information from the periphery to the cerebellum via the olive, the most comprehensively studied are those that travel in the ventral funiculus (VF) and dorsal funiculus (DF) of the spinal cord. The VF-spino-olivo-cerebellar pathway (VF-SOCP) involves a direct projection from cells of origin in the spinal cord to the contralateral inferior olive (e.g., Armstrong and Schild, 1980; Swenson and Castro, 1983; Molinari, 1984). By contrast, the DF-SOCP is indirect, and includes primary afferent projections from dorsal root ganglia cells as well as spinal cells of origin of the postsynaptic dorsal column (PSDC) that synapse in the dorsal column nuclei (DCN) which, in turn, sends projections to the contralateral olive (e.g., Ekerot and Larson, 1979a,b).

Both the DF- and VF-SOCP are functionally organized into multiple subpaths that terminate in different cerebellar cortical zones (e.g., Oscarsson and Sjolund, 1977b; Ekerot and Larson, 1979a). For example, signals arising from the ipsilateral hindlimb conveyed via the two SOCPs have a convergent site of precerebellar relay in the lateral half of the rostral dorsal accessory olive (rDAO; Armstrong et al., 1982; Gellman et al., 1983; Matsushita et al., 1992) before terminating as climbing fibers in the hindlimb-receiving parts of the C1 and C3 zones in the anterior and posterior lobes of the cerebellar paravermis (Oscarsson, 1969; Cooke et al., 1972; Berkley and Hand, 1978; Oscarsson and Sjolund, 1977a,b; Ekerot and Larson, 1979a).

The cerebellar paravermis is widely acknowledged as critically involved in the control of reflex and voluntary limb movements (for review, see Bloedel and Bracha, 1995; Apps and Garwicz, 2005) but the significance of why the same paravermal regions receive climbing fiber signals relayed via both the “direct” VF-SOCP and the “indirect” DF-SOCP remains unknown. As a first step, it is important to have knowledge of the underlying neural pathways involved. Previous single-tracer studies of the spinal cells of origin of the DF- and VF-SOCPs suggest that although the two populations are largely separate, there are potentially overlapping territories in the medial dorsal horn of the spinal cord (Rustioni, 1973,1977; Angaut-Petit, 1975; Rustioni et al., 1979; Rustioni and Kaufman, 1977; Armstrong et al., 1982; Giesler et al., 1984; Molinari, 1984). This raises the possibility that either: 1) a common population of spinal neurons exists that transmits information to rDAO both directly via the VF-SOCP and indirectly via the DF-SOCP; or 2), that these pathways arise from separate, but partly intermingled populations of spinal neurons which form the anatomical basis of a functional independence of the two ascending pathways. The possibility also exists that there are important differences between species in the organization of these ascending paths. In particular, the majority of previous studies have been performed in cats, so the present study updates knowledge in a species that is more widely used in current research.

The aim of the present experiments was therefore to use a double retrograde tracer strategy in rats to map, in the same animal, the distributions of single- and double-labeled spinal projection neurons following injections of bidirectional tracers into hindlimb-receiving components of the rDAO and the DCN. The results reveal that in rats the two pathways arise from spatially distinct populations of spinal neurons, providing the neural substrate for functional independence prior to convergence at the level of the olive.

## MATERIALS AND METHODS

### Surgery

All experimental procedures were carried out in accordance with the UK Animals (Scientific Procedures) Act 1986 and approved by the University of Bristol Animal Welfare and Ethical Review Body. Experiments were carried out on a total of 27 adult male Wistar rats anesthetized with a ketamine/medetomidine mix (60mg · kg^−1^/250μg · kg^−1^; i.p. Pfizer Animal Health, UK). Supplementary intraperitoneal doses of anesthetic were given, as required, to maintain a surgical plane of anesthesia. Rats were placed in a stereotaxic frame with the head initially tilted downward to allow access to the dorsal surface of the caudal brainstem. If necessary, the ventral portion of the occipital bone was removed to allow a clear view of obex.

Using a pneumatic picopump (WPI, UK) attached to a glass micropipette (tip diameter 50 μm), 5 × 50–100 nl (maximum of 500 nl) injections of one color of the retrograde and anterograde tracer (i.e., green or red, see below) were delivered just below the surface of the brainstem to make a unilateral, rostrocaudally oriented series of injection sites into GN, 1–1.5 mm caudal from obex. This region of GN has previously been shown to be the primary site of origin of the gracilo-olivary projection and thus a key relay in the hindlimb component of the C1 and C3 zone DF-SOCP (Molinari, 1984).

In the same animal, but with the head in the horizontal skull position (incisor bar at −3.3 mm; Paxinos and Watson, 2005), a maximum of 2 × 50–100 nl injections of a different color of a solution containing both anterograde and retrograde tracer was delivered contralateral to the GN injection into lateral rDAO to study the hindlimb C1 and C3 zone VF-SOCP (AP: obex; ML: 1.4 mm; DV: 3.8 mm; at a 37° angle; cf. Cerminara and Rawson, 2004). For each olivary injection, the micropipette was left in situ for 10 minutes postinjection, to minimize leakage of the tracer along the dorsoventral track of the pipette. After all the tracer injections were made, the brainstem was covered with gelfoam and the overlying neck muscles and skin sutured in layers. Anesthesia was reversed with a subcutaneous injection of atipamezole (1 mg · kg^−1^, s.c. Pfizer Animal Health, UK).

### Anterograde and retrograde tracers

A solution containing one color of retrograde and anterograde tracer material was used to allow both retrograde analysis of spinal projection neurons and anterograde verification of the injection site. The mixture consisted of an undiluted suspension of green or red fluorescently tagged latex microspheres (Retrobeads, LumaFluor, Naples, FL), combined with an anterograde tracer of a matching color (20% solution of fluorescent dextran amines; Fluoro-Ruby or Fluoro-Emerald; Molecular Probes, UK).

### Retrobead double cell labeling experiments

Previous studies have shown that the two colors of Retrobeads, when used on their own, produce unambiguous double retrograde cell labeling (Apps and Ruigrok, 2007). However, in the present experiments it was found that the dextran amines were also transporting to some extent in the retrograde direction and thereby interfered with the visualization of double-labeled cells, leading potentially to false positives (Schofield et al., 2007). To minimize the possibility of false-positive double-labeled cells, a second series of experiments was therefore conducted, where a further six animals received double tracer injections of red and green Retrobeads alone, with one color injected into GN, and the other color injected into rDAO. Injection procedures were identical to those detailed above.

In one additional control animal, a 1:1 mixture of red and green Retrobeads was injected into GN using the coordinates described above to determine whether the tracers were transported with equal efficiency. Retrogradely labeled cells in the dorsal horn of the spinal cord from three sections per L3–L5 segment were sampled.

### Histological processing

Following a 7–10-day recovery period, the animals were reanesthetized (Propofol, bolus, i.v., AstraZeneca, UK) and perfused transcardially with 0.9% heparinized NaCl followed by 4% paraformaldehyde. The brain, spinal cord, and, in some experiments, dorsal root ganglia (DRG) L4 and L5 were removed, postfixed for 24 hours in 4% paraformaldehyde, allowed to sink in cryoprotectant (30% sucrose in phosphate buffer [PB]), and stored at 4°C. All sections were cut into 40-μm sections using a freezing microtome. The cerebellum was separated from the brainstem and cut sagittally, while the brainstem and spinal cord were cut in the transverse plane. Spinal cord tissue from segments L3–L5 was collected as two series; cerebella and brainstem sections were split into three series.

**Figure 1:**
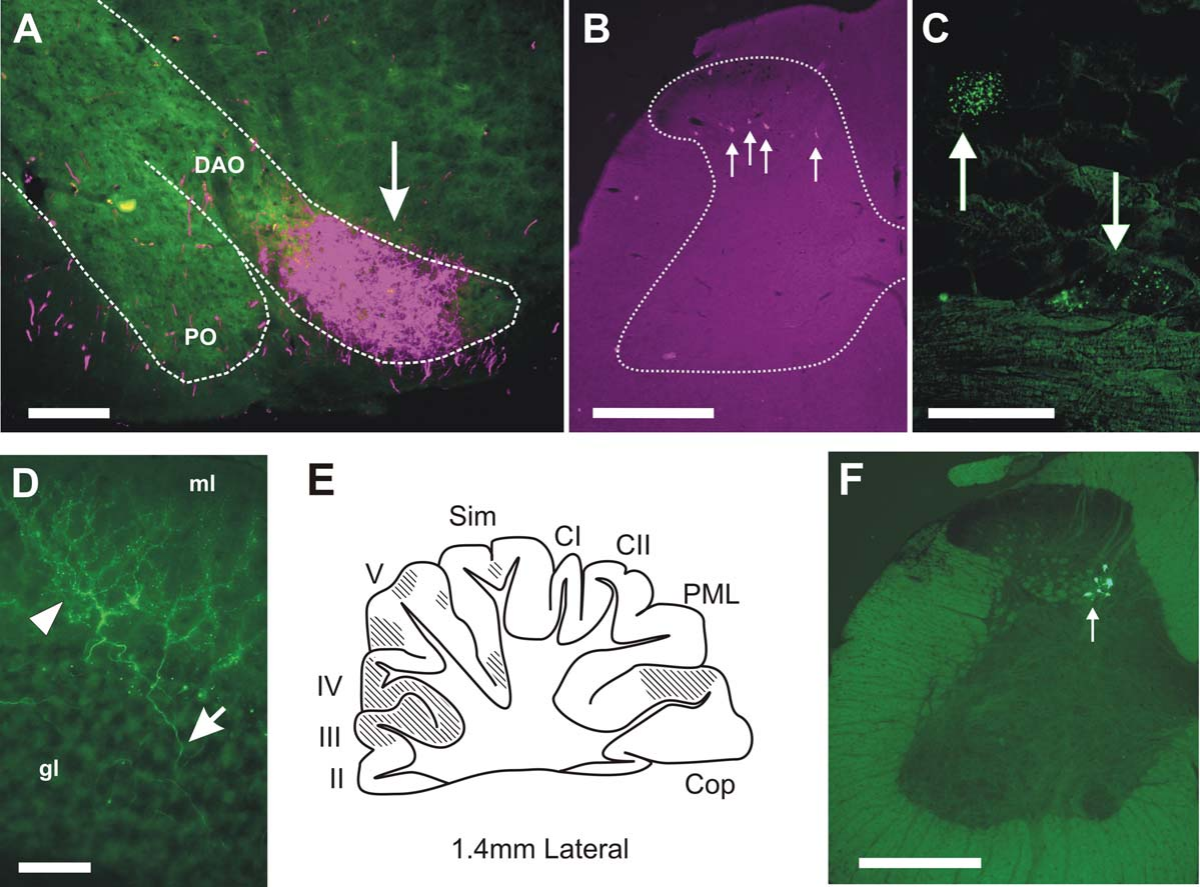
Photomicrographs of anterograde and retrograde labeling. A: An example of anterogradely labeled terminal fibers (arrow) in contralateral rostral dorsal accessory olive (DAO) as a result of injection of bidirectional tracer into the GN (case CFP78). PO, principal olive. B: Low-power photomicrograph of retrogradely labeled neurons (arrows) in lumbar segment L3 arising from an injection into the GN (case CFP104). Approximate outline of spinal cord gray matter is shown by dotted line. C: High-power photomicrograph of retrogradely labeled cells (arrows) in the ipsilateral L4 dorsal root ganglion, following injection of retrograde tracer into GN (case CFP78). D: High-power photomicrographs of anterogradely labeled climbing fibers in contralateral cerebellar cortex arising from injection of bidirectional tracer into rDAO (case CFP87). Arrowhead shows climbing fiber in molecular layer (ml) with arrow pointing to climbing fiber stem axon in granule cell layer (gl). E: Standard sagittal outline of the cerebellar cortex 1.4 mm lateral from the midline with hatching to indicate location of anterogradely labeled climbing fibers after an injection into DAO in case CFP83. II–V, cerebellar lobules II–V; CI, crus I; CII, crus II; Cop, copula pyramidis; PML, paramedian lobule; Sim, lobulus simplex. F: Low-power photomicrograph of retrogradely labeled spinal neurons (arrow) in lumbar segment L3 following injection of retrograde tracer in DAO in case CFP104. Scale bars = 200 μm in A,C; 500 μm in B,F; 50 μm in D.

Sections were mounted onto gelatin-coated slides and analyzed using a Zeiss Axioskop II Mot epifluorescent microscope (Oberkochen, Germany) fitted with a 100W-mercury UV light source and an Axiocam digital camera. Red fluorescent labeling produced by Fluoro-Ruby and/or red fluorescent Retrobeads was visualized using filter set number 15 (dichroic mirror [DM] 580 nm; band pass [BP] 546/12 nm; long pass [(LP] 590 nm). Green fluorescent labeling (Fluoro-Emerald and/or green fluorescent Retrobeads) was visualized using filter set number 9 (DM 510 nm; BP 450–490 nm; LP 515 nm). Photomicrographs were adjusted for brightness and contrast with Adobe PhotoShop (San Jose, CA) or Corel Photopaint (Ottawa, Canada) and they were assembled in plates with Adobe Illustrator or Corel Draw. Red color was converted to magenta for the benefit of color-blind readers (e.g., Fig. 1).

### Analysis of retrograde and anterograde labeling

The extent of the injection sites was plotted onto standard transverse maps of the medulla (Paxinos and Watson, 2005). Analysis of retrogradely labeled cells in the spinal cord was confined to lumbar segments L3–L5 because in rat these segments receive the majority of peripheral afferents from the hindlimb, particularly those arising from the hindpaw (Grant and Robertson, 2004). An estimate of the numbers and distributions of retrogradely labeled neurons (single-labeled red or green and, where appropriate, double-labeled red and green) in alternate spinal cord sections was made and their distribution mapped onto standard spinal cord maps (Paxinos and Watson, 2005). Only labeled cell bodies were counted; labeled dendrites or cell fragments were disregarded. As the sections were 40 μm thick and only every other spinal cord section was counted, a cell body that was split across three sections would need to have a cell body that was in excess of 80 μm. A previous study has indicated that in rat lumbar spinal cord, cell bodies do not exceed 30 μm in diameter, including in the rostrocaudal plane (Molander et al., 1984); therefore, it is very unlikely that our analysis overestimated counting of cells. Instead, the sampling was likely to underestimate the true numbers of labeled cells. We therefore used the Abercrombie correction (Abercrombie, 1946) to adjust the counts accordingly. The correction factor applied was as follows:

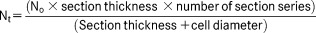


Where Nt and No are the true and observed numbers of cells, respectively. The mean diameter of a spinal cord neuron in the lumbar segment was taken from individual lamina reported by Molander et al. (1984). These ranged from 5 μm in laminae I–III and X to 30 μm in lamina X. Thus, in the present experiments:

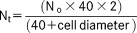
where the Abercrombie correction factor applied for different laminae varied from 1.14 to 1.78.

All mean cell counts in the Results are Abercrombie-corrected.

To generate “cluster” diagrams (Fig. 3), outlines were drawn around groups of three or more cells that were less than 0.5 mm apart and the outlines from all cases overlaid. Analysis of anterograde labeling in the cerebellar cortex was carried out on every third sagittal section of the cerebellum. The regional distribution of any climbing fiber terminal labeling resulting from injection of anterograde tracer into rDAO was denoted by hatching (e.g., Fig. 1E). The same method was used to chart the extent of terminal labeling in rDAO resulting from injection of anterograde tracer into GN (not shown). To confirm direct projections to GN, in four animals the DRG were also recovered and cut longitudinally (10 μm) on a cryostat and examined for retrograde cell labeling of DRG neurons.

In different experiments either the red or green mixture of tracers was injected into each target. However, for clarity of presentation, and to allow data from multiple cases to be pooled, all injections are illustrated as though red tracers were injected into the left GN and green tracers were injected into the right rDAO.

To quantify the distribution of retrogradely labeled neurons in the spinal cord, each single- or double-labeled neuron was assigned to a particular spinal cord lamina (laminae I–II, III, IV, V–VI, VII–VIII, IX, X) or spinal nucleus (lateral spinal nucleus, LSN). As the injections into rDAO resulted in two subpopulations within laminae V–VI of segment L3 (see Results), this grouping was further divided into two: a lateral and medial region. The total number of neurons localized in each group in lumbar segments L3, L4, and L5 was calculated, and the data from all experiments were pooled and then compared using one-way analysis of variance (ANOVA) and Tukey’s multiple comparisons test, using the statistical analysis software GraphPad Prism v. 6.0 (San Diego, CA).

## RESULTS

A total of 19 animals were used to study the spatial distribution of spinal cord cells in the lumbar enlargement (L3–L5) that project to hindlimb-receiving parts of the dorsal column nuclei (GN) or inferior olive (lateral rDAO). In each animal one color of bidirectional tracer mixture was injected into GN and the other color injected into lateral rDAO. Injections of tracer into GN were always contralateral to injections made into rDAO. No differences were found in the numbers of spinal neurons labeled from transport from red or green injection sites.

### Injection sites in GN and rDAO

Because a mixture of anterograde and retrograde tracer was used in each injection, it was possible to gauge the extent to which the injection site in GN or rDAO was on target for the hindlimb component of the DF-SOCP and VF-SOCP, respectively. GN injections were judged to be on target if: 1) the injection site was centered on the transitional zone of GN (1–1.5 mm caudal to obex; Molinari 1984); 2) anterogradely labeled terminals were located in the lateral half of rDAO (arrow, Fig. 1A); and 3) retrogradely labeled cell bodies were located in the spinal cord (arrows, Fig. 1B). For all GN injections (*n* = 19) these criteria were met, although the full extent of anterograde labeling in rDAO could not be mapped as it was generally obscured, at least to some extent, by the injection site of the other tracer mix in rDAO. On average, 246 ± 35 (mean ± SEM) spinal neurons were labeled in lumbar segments L3–L5 after injections into GN. In four animals the dorsal root ganglia (DRG) for spinal segments L4 and L5 were also studied, and in each case retrograde cell labeling (due to transport in the dorsal column pathway) was also found (arrows, Fig. 1C).

Injections into rDAO were judged to be on target if: 1) they were centered on the lateral part of rDAO; 2) anterogradely labeled climbing fibers were present, but not necessarily confined to, “hindlimb-receiving” areas of the cerebellar cortex (copula pyramidis and paravermal lobules I–IV (including presumably the hindlimb parts of the C1 and C3 zones; Atkins and Apps, 1997; Eisenman, 1981; Jorntell et al., 2000; Fig. 1D,E); and 3) retrogradely labeled cell bodies were located in the spinal cord (Fig. 1F). These criteria were satisfied in 13 of the 19 cases. On average, 200 ± 41 (mean ± SEM) spinal neurons were labeled in lumbar segments L3–L5 after injections into rDAO. This value was not significantly different from the mean number of spinal neurons labeled after injections into GN (*P* = 0.40, Welch’s unpaired *t*-test). However, it should be noted that the possibility remains that differences in cell counts may occur within segmental levels not studied in the present experiments.

In two additional animals (cases CFP116 and 117) the injection sites were localized above the rDAO and, in common with injections into rDAO, a little tracer spread along the dorsoventral pipette track through the brainstem. In both of these cases there were a few retrogradely labeled neurons in the spinal cord (10 and 14 cells in CFP116 and CFP117, respectively, located within lamina V–VIII). No evidence of labeled climbing fibers could be found in either case, although some labeled mossy fibers were identified in CFP117 (not illustrated). The low incidence of retrogradely labeled spinal neurons in these two cases suggest that most of the retrogradely labeled neurons in the spinal cord in the 13 double tracer experiments selected for detailed analysis are likely to have originated from deposition of tracer into rDAO rather than areas immediately dorsal to it.

### Distribution of spinal cord neurons following injections into DAO and GN

**Figure 2:**
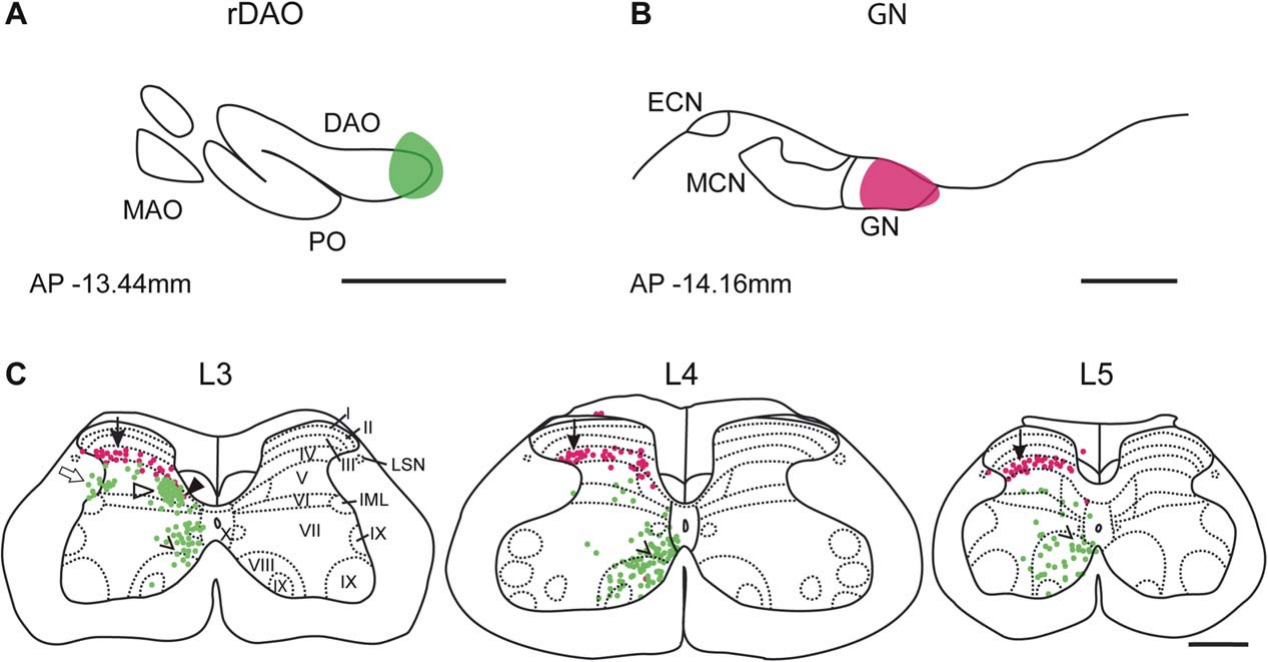
Distribution of spinal cord neurons following injections into rDAO and GN. Schematic transverse representations of injection sites in rDAO (A), and GN (B) in case CFP89. Values for each level indicate approximate AP coordinates from Bregma (Paxinos and Watson, 2005). ECN, external cuneate nucleus; DAO, dorsal accessory olive; GN, gracile nucleus; MAO, medial accessory olive; MCN, main cuneate nucleus; PO, principal olive. C: Standard transverse outlines of the lumbar spinal cord depicting the pattern of retrograde cell labeling in segmental levels L3, L4, and L5. Magenta dots denote cells labeled from the GN injection site. These are concentrated in a mediolateral band within lamina IV (arrow); green dots denote cell labeling arising from the DAO injection site. Cell labeling from rDAO form three populations: a cluster in the medial aspect of lamina V in L3 (filled arrowhead); a cluster in lateral lamina V and adjacent white matter in L3 (unfilled arrow); and a cluster in the ventromedial aspect of lamina VII/VIII in L3, L4, and L5 (open arrowhead). Each dot represents an individual retrogradely labeled cell. Spinal laminae (I–X) according to Molander et al. (1984). LSN, lateral spinal nucleus; IML, intermediolateral cell column. Scale bars = 500 μm.

An example of a double tracer experiment is shown in Figure 2. Figure 2A,B illustrate the injection sites in rDAO and GN, respectively. Typical of the material as a whole, retrograde transport from the injections into rDAO and GN labeled two almost entirely separate populations of cells in lumbar segments of the spinal cord, with no double-labeled cells found in L3–L5 (Fig. 2C). In segments L3–L5, spinal neurons labeled from the injection site in GN were located predominantly within lamina IV (arrow, Fig. 2C), representing 78% of the total GN projecting cell population, while cells retrogradely labeled from the injection site in rDAO were located mainly within three territories: 1) the medial part of lamina V in segment L3 (unfilled arrowhead, Fig. 2C), representing 20% of the total rDAO projecting cell population; 2) the lateral part of lamina V and adjacent white matter in segment L3 (unfilled arrow), representing 10.5% of the total rDAO projecting cell population; and 3) the medial part of laminae VII and VIII in L3–L5 (open arrowhead), representing 48% of the total rDAO projecting population. A small area of overlap in the two populations of single-labeled cells was present in medial lamina V (filled arrowhead, L3).

**Figure 3:**
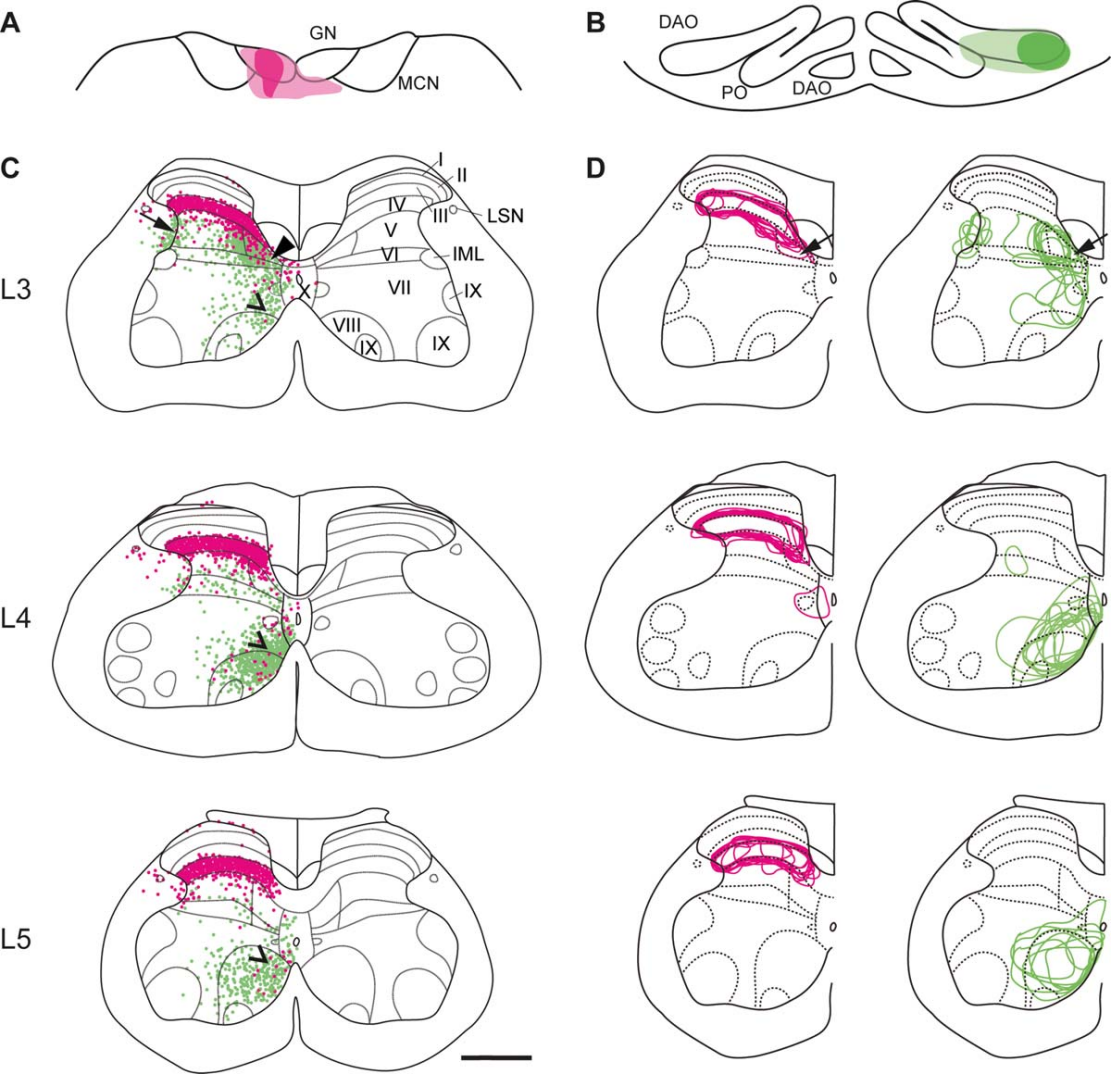
Overall distributions of spinal neurons that project to the GN and rDAO. A,B: Schematic transverse outlines of GN and DAO to show location of all injections sites considered for detailed analysis (*n* = 13). Dark shading shows core injection site area of all cases; light shading shows maximum extent of injection sites. C: Pooled data from all double bidirectional tracer experiments to illustrate the distributions of neurons retrogradely labeled from injection sites centered on GN (magenta) and rDAO (green) in lumbar segments L3, L4, and L5. Each dot indicates a retrogradely labeled cell. Three populations of cells were retrogradely labeled from the injection site in rDAO: i) Filled arrowhead indicates location of cells in the medial part of lamina V in segment L3; ii) arrow indicates location of cells in lateral part of lamina V and adjacent white matter in segment L3; and iii) open arrowhead indicates the cell population in the medial part of laminae VII and VIII in L3, L4, and L5. D: Same segments showing data for each case depicted as outlines of “clusters” of neurons. Magenta outlines show regions projecting to GN, green outlines show regions projecting to rDAO. See Materials and Methods for further details. Arrow indicates main region of overlap of magenta and green outlines. Scale bar = 500 μm.

To allow a detailed analysis of the distribution of GN-projecting and rDAO-projecting spinal neurons, the maps of cell labeling from cases where injections were judged to be centered on GN and rDAO were pooled (*n* = 13, Fig. 3). Figure 3A,B shows the total extent of the injection sites (light shading) pooled for the 13 cases. The dark shading indicates “core” territories where the injection sites overlapped in all cases. The labeled spinal neurons in segments L3, L4, and L5 were assigned to individual lamina or spinal cord regions (laminae I–II, III, IV, V–VI, VII–VIII, IX, X, LSN; see Materials and Methods for further details).

Following injections into GN, the large majority of retrogradely labeled cells were located in lamina IV, representing 77% of the total GN projecting cell population for all cases pooled (magenta dots, Fig. 3C). The spatial localization in lamina IV is highlighted by the “cluster” analysis (Fig. 3D, see Materials and Methods for details). Some cell labeling was also present in lamina V and a few scattered cells were located in the LSN, and ventromedially in laminae VI–IX and lamina X.

Neurons retrogradely labeled after injections into rDAO (green dots, Fig. 3C) displayed a more complex pattern of distribution that varied according to lumbar segment. The spatial differences are highlighted by the “cluster” analysis (Fig. 3D, see Materials and Methods for details). Some retrograde cell labeling was present in lamina X. However, the majority of cells retrogradely labeled from the injection site in rDAO were located mainly within three territories: i) the medial part of lamina V in segment L3 (filled arrowhead, Fig. 3C), representing 19% of the total rDAO projecting cell population of L3; ii) the lateral part of lamina V (including adjacent white matter) in segment L3 (arrow, Fig. 3C), representing 3.5% of the total rDAO projecting cell population of L3; and iii) the medial part of laminae VII and VIII (open arrowhead), representing 36% of the total rDAO projecting cell population). Populations (i) and (ii) were only present in L3, while population (iii) was present throughout L3, L4, and L5. In most cases a small area of overlap of the two populations (GN and rDAO) of single-labeled cells occurred medially in lamina V at segmental level L3 (arrow, Fig. 3D). The number of GN projection neurons in this area of overlap accounted for less than 5% of the total cell population. For consideration of cell double labeling, see below.

**Figure 4:**
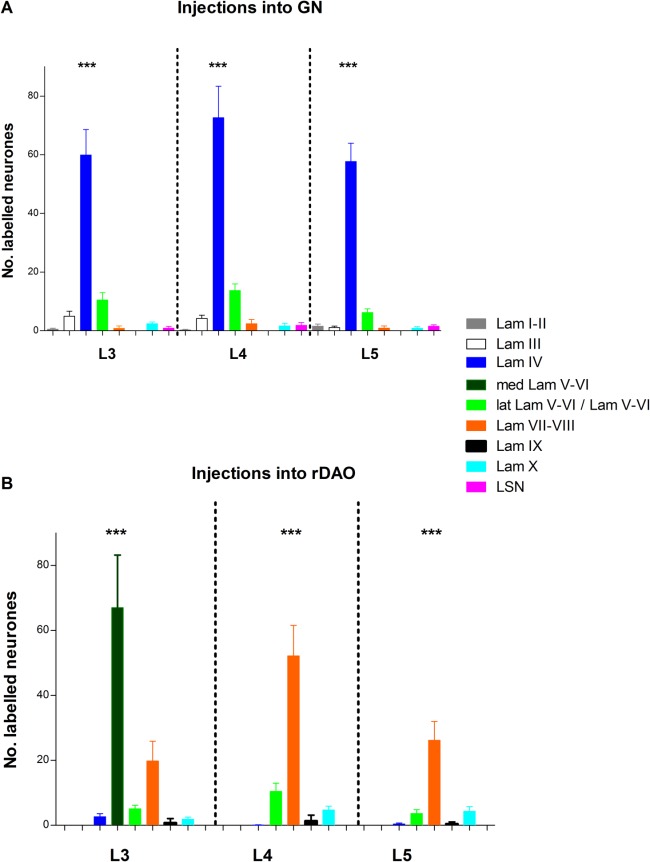
Quantitative analysis of lamina distribution of L3–L5 spinal projection neurons. A: Histogram plots mean cell counts per spinal laminae and spinal nuclei for segmental levels L3–L5 arising from injections into GN. B: Histogram plots mean cell counts per spinal laminae and spinal nuclei for segmental levels L3–L5 arising from injections into rDAO. Counts are Abercrombie-corrected (see Materials and Methods for further details). Data are plotted as mean ± SEM, *n* = 13 double tracer experiments. Statistical comparisons (one-way ANOVA and Tukey’s multiple comparisons test) were made between laminae at each segmental level, ***P < 0.001. LSN, lateral spinal nucleus.

Figure 4 is a quantitative analysis of cell counts in different spinal regions in the three lumbar segments. For GN projection neurons, significantly more labeled neurons were located within lamina IV than in any other spinal region throughout segments L3–L5 (dark blue bars, Fig. 4A, L3: F_(7,96)_ = 39.11 *P* < 0.0001; L4: F_(7,93)_ = 37.71, *P* < 0.0001; L5: F_(7,93)_ = 73.97, *P* < 0.0001, *n* = 13; one-way ANOVA and Tukey’s multiple comparison test). By comparison, for rDAO projection neurons, in segment L3 significantly more labeled neurons were located in medial laminae V–VI, compared to every other group (dark green bar, Fig. 4B, F_(8,108)_ = 14.07, *P* < 0.0001, *n* = 13; one-way ANOVA and Tukey’s multiple comparison test). While in segments L4 and L5, there were significantly more rDAO projection neurons localized to laminae VII–VIII than any other group (orange bars Fig. 4B, L4: F_(7,96)_ = 27.49, *P* < 0.0001, L5: F_(7,96)_ = 17.73, *P* < 0.0001, *n* = 13; one-way ANOVA and Tukey’s multiple comparison test).

### Lack of double-labeled neurons following retrograde tracer injections into GN and DAO

**Figure 5:**
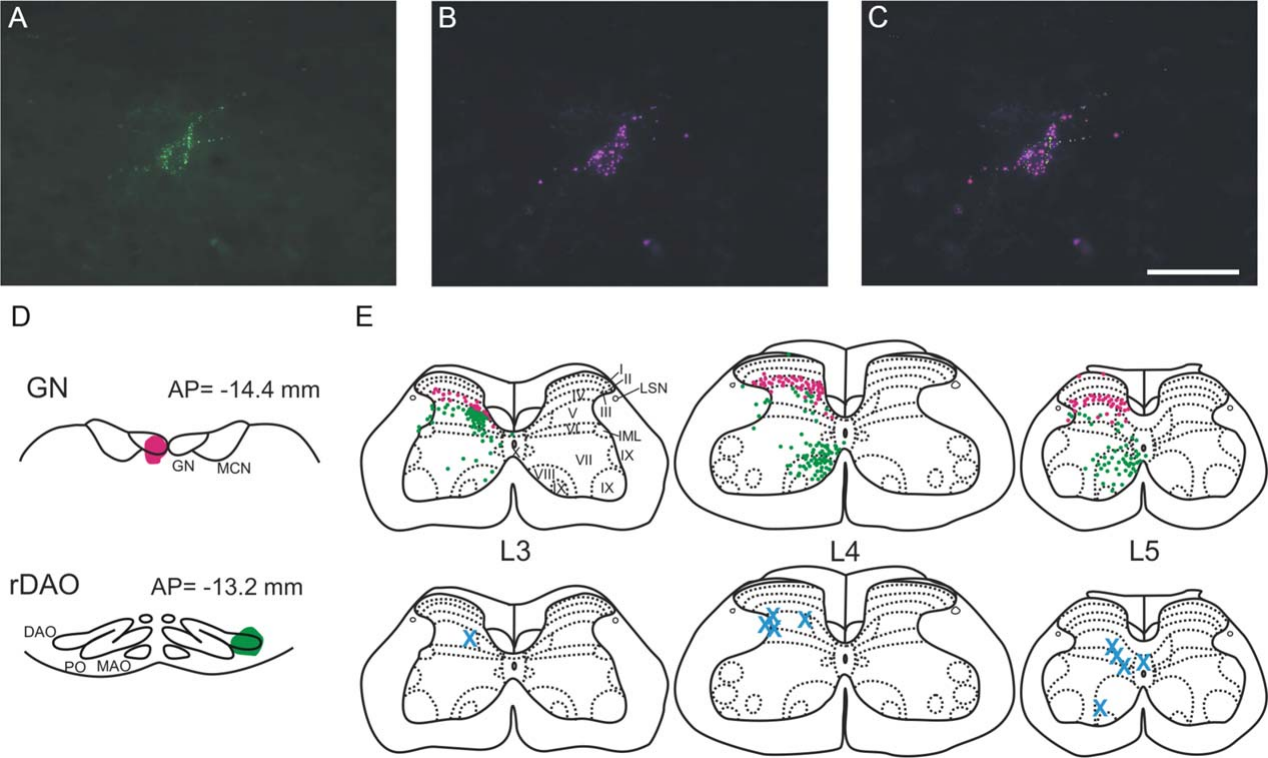
Lack of double-labeled neurons following retrograde tracer injections into GN and DAO. A–C: High-power photomicrographs showing an example double-labeled cell in case CFP111 (A, field viewed for green fluorescence; B, same field viewed for red fluorescence; C, both views combined). D: Injection sites in GN and rDAO. E: Upper panel, distribution of magenta and green single-labeled cells in spinal cord segmental levels L3–L5. Each dot represents an individual labeled cell. Lower panel, distribution of double-labeled cells (blue crosses). Same abbreviations as in Fig. 2. Scale bar = 50 μm in C (applies to A,B).

In six additional double tracer experiments, Retrobeads were used as retrograde tracers to reliably identify double-labeled cells (see Materials and Methods for further details). Figure 5A,B illustrate retrogradely labeled cells in the spinal cord from injection sites in GN and rDAO. In all six experiments there was a close correspondence between the spatial distribution of single-labeled cells in the spinal cord and the overall pattern obtained for the more extensive bidirectional tracer experiments (compare upper row Fig. 5E with Figs. 2 and 3). Only 10 double-labeled spinal neurons were ever found (lower row Fig. 5E, blue crosses), representing just 1.4% and 0.7%, respectively, of the total single tracer cell populations arising from GN and rDAO injections. The double-labeled cells were located mainly in laminae V/VI in spinal segments L4 and L5 in regions where there was also some overlap of the two single-labeled cell populations.

To control for the possibility that the low numbers of double-labeled cells found may be due to limitations of the methods used, a control experiment was carried out in which a 1:1 mix of red and green Retrobeads was injected into the gracile nucleus (see Materials and Methods). In a sample of sections from lumbar segments L3–L5 a total of 54 red and 53 green retrogradely labeled cells were found. The percentage that were double-labeled was 96.4% and 94.6%, respectively, demonstrating that both tracers had very similar transport characteristics. The low number of doubles found in the experiments was therefore likely to be a reliable result.

## DISCUSSION

The present study used a dual tracer strategy in rat to allow direct comparison of the spatial distributions of spinal cord cells of origin of the hindlimb components of the DF- and VF-SOCPs. The key findings were that: 1) the two groups of projection neurons were found to occupy mainly separate areas of the lumbar spinal cord; 2) there is a small area of overlap in the medial half of lamina IV–V in L3 but in this region of overlap, the two populations of cells are intermingled with very few double-labeled cells; and 3) there are differences between rat and cat in the spatial organization of spino-olivary paths.

### Comparison with previous anatomical studies of spino-olivary projections

In the current study, spinal neurons in the lumbar enlargement that project directly to rDAO (and are therefore likely to include cells of origin of VF-SOCPs targeting the hindlimb parts of the cerebellar cortical C1 and C3 zones) were located in three spatially distinct populations: i) in the medial aspect of laminae V–VI; ii) in the ventromedial ventral horn (laminae VII–VIII); and iii) in the lateral aspect of lamina V.

Previously, the most detailed anatomical study (in the cat) of lumbar spino-olivary pathways targeting the DAO also concluded there were three distinct populations of neurons: a group in the dorsal horn (central part of lamina V), a group in the ventromedial ventral horn, and a third group in the white matter immediately adjacent to the dorsal horn (Molinari, 1984). It was unclear if a fourth group in the intermediate gray matter (lamina VI/VII) was the result of retrograde transport from the olive or the neighboring reticular formation. Our findings in rat are in general agreement with these results, suggesting similar principles of organization of spino-olivary projections across species (Brown et al., 1977; Armstrong and Schild, 1980; Richmond et al., 1982; Armstrong et al., 1982; Molinari, 1984; McCurdy et al., 1998). However, our results also suggest that the group of projection neurons in lamina V are closer to the midline than previously described. This is consistent with a previous study in rat (Swenson and Castro, 1983) using horseradish peroxidase (HRP) as a retrograde tracer, where two populations of spino-olivary cells were reported: one group dorsomedially and a second group ventromedially, although these were reported only at more rostral segments of the lumbar cord (L1–L3). Swenson and Castro (1983) failed to identify a third population of projection neurons in lateral lamina V. This and the absence of cell labeling in more caudal lumbar segments in their study may be attributed to their use of a less sensitive retrograde tracer.

It is also of interest to compare the present findings with those of Armstrong et al. (1982), who correlated the location of injection sites centered on rostral and caudal parts of the olive in cat with the known electrophysiological properties of subpaths within the VF-SOCP and their different zonal targets in the cerebellar cortex (Oscarsson and Sjolund, 1977a–c). Armstrong et al. (1982; see also Molinari 1984,1985) suggested that cells in the lumbosacral cord retrogradely labeled in the dorsal horn, intermediate gray, and in the ventromedial ventral horn were spinal relays for the hindlimb components respectively of the C1 and C3 zone VF-SOCP (relayed via rDAO), the B zone VF-SOCP (relayed via caudal DAO), and the A zone VF-SOCP (relayed via the caudal medial accessory olive, MAO). The present results challenge this interpretation and instead support the view that in rats the different populations of cells in the lumbar cord (except perhaps cells in the intermediate gray) all provide projections to rDAO and are therefore spinal relays for the hindlimb C1 and C3 zone VF-SOCP. The possibility remains to be tested whether the same regions of spinal cord also provide projections to other parts of the olive involved in VF-SOCP subpaths that target other cerebellar cortical zones.

### Comparison with previous anatomical studies of the postsynaptic dorsal column projection

Previous retrograde tracer studies in rats have identified a mediolateral band of cells in laminae III/IV and to a lesser extent cells in lamina X as the source of the PSDC projection to GN (de Pommery et al., 1984; Giesler et al., 1984). The present results are in good agreement with this but indicate that in lumbar segments L3–L5, lamina IV is by far the largest source of the projection in rats. Some retrogradely labeled cells were also noted in lamina V. This is similar to the location of cells of origin of the PSDC in cat which has been reported as originating primarily from ventral parts of nucleus proprius (i.e., laminae IV/V; Rustioni and Kaufman, 1977; Bennett et al., 1983; Enevoldson and Gordon, 1989). In the present study some scattered cell labeling was also found in the lateral spinal nucleus and in other laminae, notably laminae VII and VIII. Giesler et al. (1984) noted in rat that spread of injectate into the reticular formation underlying the DCN resulted in cell labeling in deeper laminae. This raises the possibility that in our experiments the diffuse cell labeling found in these additional spinal cord areas was due to some spread of the GN injection sites ventrally. For example, spinoreticular projections originate from cells in deeper laminae (Chaouch et al., 1983), while the nucleus of the solitary tract, which lies immediately below the GN, receives projections from the lateral spinal nucleus (e.g., Menétrey and Basbaum, 1987; Esteves et al., 1993; Gamboa-Esteves et al., 2001; Gamboa-Esteves et al., 2004).

### Spatial separation of GN and DAO projection neurons in the spinal cord

A unique aspect of the present study was that the spinal origins of direct and indirect SOCPs could be compared directly in the same animal. Fluorescent microspheres were used as retrograde tracers, which are highly effective as double retrograde tracers (Katz et al., 1984; Hudson and Lumb, 1996; Apps and Garwicz, 2000; Apps and Ruigrok, 2007; Schofield et al., 2007; Herrero et al., 2012). It therefore seems unlikely that the small number of double-labeled cells detected was due to any limitations of the methods used. This is supported by the finding that in our control experiment we obtained nearly 100% double-labeled cells when the Retrobeads were mixed in a 1:1 ratio. Additionally, the two single-labeled cell populations were located mainly in spatially separate areas of the lumbar cord with only very limited overlap.

### Concluding comments

The hindlimb C1 and C3 zone VF- and DF-SOCPs share a number of physiological features in common. Namely, climbing fiber projection to the same cerebellar cortical zones, large receptive fields, and they both exhibit extensive multimodal convergence of peripheral afferents (low and high threshold cutaneous and group II/III muscle afferents; Oscarsson, 1969; Oscarsson and Sjolund, 1977a–c; Ekerot and Larson, 1979a,b). This suggests that both SOCPs are unlikely to forward specific sensory information from the periphery, for example, relating to the stimulation of a particular receptive field, but instead forward a highly integrated signal concerning primary afferent influence on spinal circuits (Oscarsson, 1967,1968,1969). However, the two pathways also display a number of important differences. The C1 and C3 zone VF-SOCPs are monosynaptically activated by peripheral sensory inputs and therefore represent the most direct of all SOCPs, with only a disynaptic link between the periphery and climbing fiber terminals in the cerebellar cortex (Sjolund, 1978). In addition, transmission in these pathways is little influenced by descending control systems (Andersson and Sjolund, 1978; Sjolund, 1978), i.e., this channel offers a potentially “secure route” by which information from the spinal cord can influence cerebellar circuitry. By contrast, the C1 and C3 zone DF-SOCPs include cells of origin of the PSDC and have additional brainstem relays within DCN and are subject to substantial descending control at spinal and supraspinal levels (Andersson, 1984; Noble and Riddell, 1989). This information channel therefore has the potential to be modulated during behavior, as seen in a range of task-dependent settings for DF-SOCP projections to the forelimb C1 and C3 zones (e.g., Apps et al., 1995,1997; Apps and Lee, 2002).

With regard to the PSDC, the experimental approaches used in the present study do not allow us to determine whether or not there are two independent components—one that forwards information to the thalamus, the other that forwards information to the olive, and then on to the C1 and C3 zones of the cerebellar cortex. Future studies are required to establish whether this important distinction exists, since this is of considerable functional significance in terms of identifying the level of the neuraxis at which sensory and motor systems become segregated.

In summary, the present study demonstrates that the direct VF-SOCP spinal projection to rDAO arises from spatially distinct areas of the spinal cord compared to the indirect DF-SOCP. The spatial separation of these two populations of neurons permits transmission and/or regulation of functionally independent channels of information to olivocerebellar climbing fibers terminating in the hindlimb C1 and C3 zones. Given that the PSDC has important somatosensory functions (e.g., Willis and Coggeshall, 2004; Palecek, 2004) and is subject to descending control, this pathway may form the substrate for parallel processing of sensorimotor information prior to feeding into cerebellar circuits.
